# The effect of low-dye taping on rearfoot motion and plantar pressure during the stance phase of gait

**DOI:** 10.1186/1471-2474-9-111

**Published:** 2008-08-18

**Authors:** Kieran O'Sullivan, Norelee Kennedy, Emer O'Neill, Una Ni Mhainin

**Affiliations:** 1 Physiotherapy Department, University of Limerick, Ireland; 2 Physical Activity, Occupation and Health Research Unit, University of Limerick, Ireland

## Abstract

**Background:**

Low-dye (LD) taping is commonly used to reduce rearfoot pronation. No studies have previously investigated the effectiveness of LD taping using both plantar pressure distribution (F-Scan) and 3-D (CODA) analysis of rearfoot motion.

**Methods:**

20 healthy subjects with a navicular drop test exceeding 10 mm participated in the study. T tests were used to determine whether significant (p < 0.05) differences in plantar pressure and rearfoot motion occurred with LD taping.

**Results:**

LD taping resulted in statistically significant increases in peak plantar pressure in the lateral midfoot (p = 0.000), along with significant decreases in pressure in the medial forefoot (p = 0.014), and the medial (p = 0.000) and lateral hindfoot (p = 0.007). No significant changes occurred in the medial midfoot (p = 0.794) or lateral forefoot (p = 0.654). When assessed using motion analysis, taping resulted in a statistically significant decrease in rearfoot pronation (p = 0.006), supination (p = 0.025) and total rearfoot range of motion (p = 0.000). The mean rearfoot position during stance was not significantly different however (p = 0.188).

**Conclusion:**

LD taping is associated with alterations in peak plantar pressure in the midfoot and forefoot that indicate reduced pronation with LD taping. However, LD taping appears to reduce both pronation and supination in the rearfoot, rather than simply reducing pronation, when assessed using 3D motion analysis. Therefore, it would appear that LD taping does indeed reduce pronation, by restricting rearfoot motion in general, rather than pronation specifically. The degree of change observed with LD taping was however very small, and further research is needed to clarify the clinical significance of these initial findings.

## Background

Pronation is a normal component of the stance phase of gait, however excessive pronation, when the rearfoot remains pronated beyond the midstance phase of gait [1], may cause excessive myofascial and soft tissue stress [2]. Low-dye (LD) taping is commonly used by physiotherapists in the treatment of lower limb symptoms related to altered or excessive pronation [3,4], or to help decide if orthotics may be indicated [5]. LD taping is suggested to limit foot pronation by raising the medial longitudinal arch and controlling the amount of rearfoot pronation occurring [6,7].

The effectiveness of LD taping has been examined in many different ways, including static and dynamic measures. Static measures include assessing vertical navicular height (VNH) and the navicular drop test (ND) [6,8]. Using these measures, it appears that LD taping increases VNH and reduces ND in stance [8,9], implying a short-term reduction of pronation with LD taping. Dynamic analysis of the effect of anti-pronation taping on foot motion and alignment has been less commonly used, even though studies have questioned the actual validity of static measures in predicting dynamic foot function [1]. A previous study [7] used two-dimensional (2D) video analysis to measure the pronation angle of the foot with and without tape. Their results did not show any significant difference in dynamic pronation under each of the conditions. In contrast, other authors [10] who also used 2D video analysis, found the arch height ratio to increase, which indicated reduced pronation, in overpronated subjects after the application of LD taping whilst walking. There are no published trials examining the effect of LD taping using three-dimensional (3D) motion analysis. Previous 3D analysis of the effects of soft foot orthotics on overpronation however found significantly reduced overall rearfoot motion when the orthotics are used, and not just reduced pronation [11]. While there has been research into the effect of LD taping on rearfoot pronation, the effect of LD taping on rearfoot supination and overall rearfoot motion is unclear.

Numerous studies have used plantar pressure patterns as an indirect measure of foot pronation during walking, as it has been assumed that plantar pressure distribution reflects rearfoot position [12]. The existing evidence suggests that LD taping reduces pronation, as indicated by shifts in midfoot pressure from medial to lateral, as well as changes in forefoot and hindfoot forces [3,12]. Many previous trials examining plantar pressure however did not individually calibrate the sensors for each individual, did not allow subjects wear their usual footwear, and may not have investigated truly normal gait due to subjects having to step onto a sensing platform [3,12].

The purpose of this study was therefore to evaluate the immediate effect of LD taping, using 3D motion and plantar pressure distribution analysis. A population of healthy subjects with a navicular drop exceeding 10 mm were chosen to attempt to replicate the patient population who might receive LD taping.

## Methods

Ethical approval was obtained from the University of Limerick Research Ethics committee. Participants gave written informed consent prior to participation.

### Subjects

A convenience sample of 28 healthy subjects volunteered to participate in this study. An initial screening session determined if subjects had excessive pronation, using the ND test, which is a commonly used method for measuring excessive pronation in healthy individuals, and which has good intra-rater reliability [8,13]. Excessive pronation was defined as navicular drop of > 10 mm, similar to previous research [8,13,14] and all subjects were screened by one investigator. Eight were excluded as they did not have a navicular drop of greater than 10 mm. Tape allergy testing was also performed at the initial screening, for which a piece of zinc oxide tape was applied to the right ankle, and left in situ for at least 24 hours. Subjects with an adverse skin reaction (redness, rash or discomfort) to tape, with a lower limb injury in the past six months, or who were unable to walk painfree were excluded.

### Study design

A repeated measures crossover study design was used. Since the plantar pressure and 3D motion data could not be collected simultaneously due to the practical issues in using both pieces of equipment, the order of testing was structured to minimise the length of time required for testing. Therefore the sequence of testing was always as described in Table 1. This allowed each subject to be analysed using both systems separately with a requirement to be only taped once.

**Table 1:** Order of testing for both procedures, and taping condition of each.

**Order**	**Test procedure**	**Taped**
1	Plantar pressure	No
2	Plantar pressure	Yes
3	3D motion analysis	Yes
4	3D motion analysis	No

### Taping

**Figure 1:**
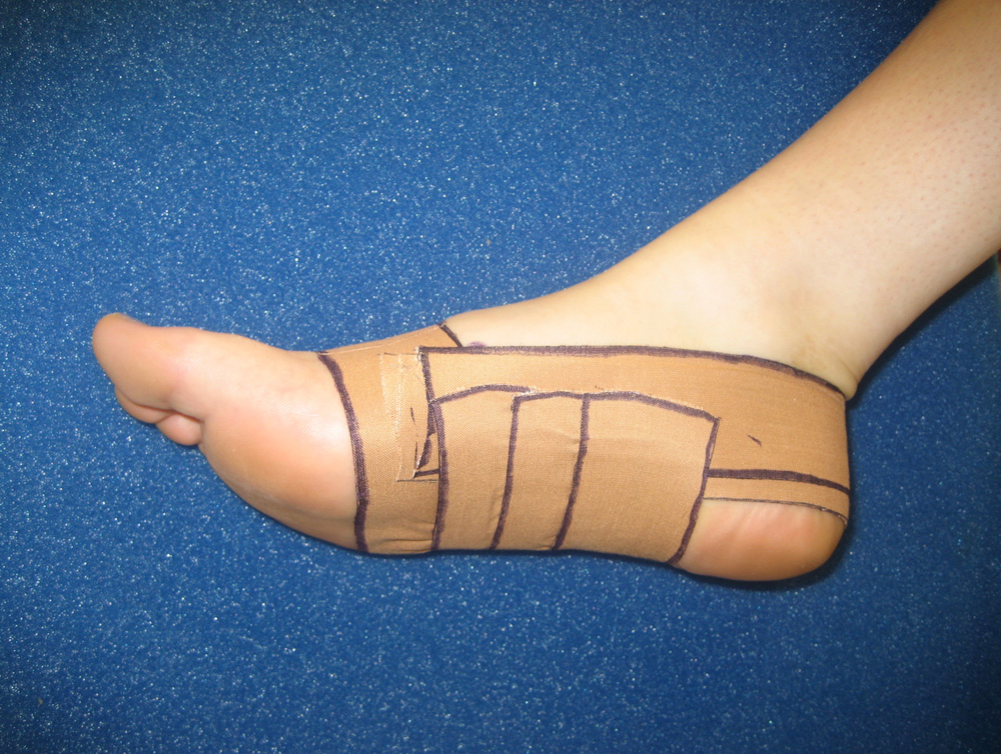
Low-dye taping technique used in the study.

LD taping was applied only to the right foot of each subject [3]. A standard LD taping technique using rigid 3.8 cm wide zinc oxide tape (Leukotape) was used, similar to other trials [3,6,12], while palpating subtalar joint neutral position (Figure 1). Feet were washed and dried in advance of taping to optimise tape adherence [5]. To enhance consistency, the same investigator applied all taping and followed a standardised protocol.

### Instrumentation: Plantar pressure Data

**Figure 2:**
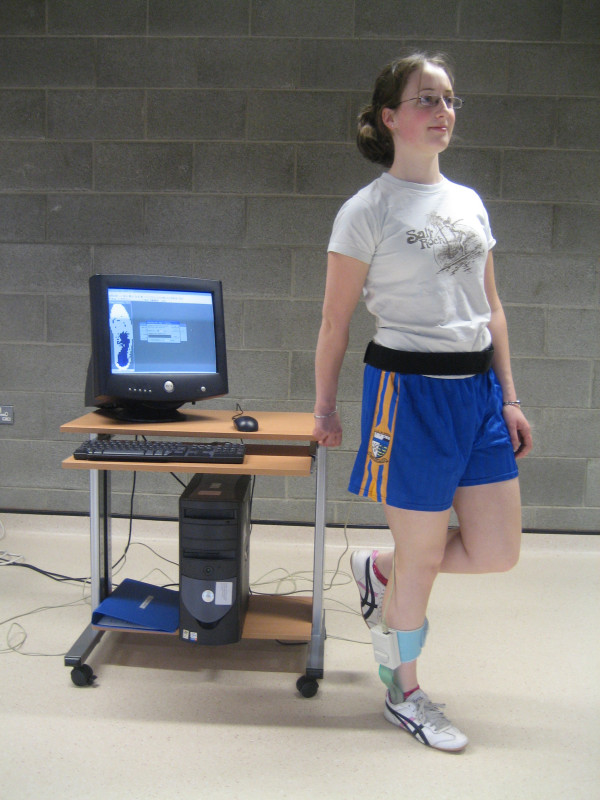
Calibration procedure for the F-scan plantar pressure system.

The F-Scan (Tekscan Inc), a computerised insole sensor system, was used to measure plantar pressure. The sensor consists of a bipedal, thin shoe insole composed of 960 individual pressure-sensing locations, providing a spatial resolution of four sensors/cm^2^. The insole uses resistance-based technology and the inner surfaces are printed with electrical circuits and in between these circuits is a semiconductive ink whose electrical resistance change inversely proportionally to the pressure applied. Studies have found that the F-Scan has fair to good reliability. Ahroni et al. [15] examined the reliability of the F-Scan in people with diabetes and found moderate ICC values of 0.755 and 0.751. Mueller and Strobe [16] examined the reliability of the F-Scan in ten normal subjects over multiple steps and reported a pearson product moment correlation coefficient of 0.933 between force platform data and F-Scan data. An experimental comparison of the Pedar system and the F-Scan by Hsiao et al. [17] also reported good reliability for both systems provided the limitations of using such measurement devices were identified and reduced where possible. The F-Scan insoles were measured for each individual's right shoe according to manufacturers guidelines. The sensor was then inserted into the subjects shoe and attached to the transducer device that is attached to a computer via a 9.25 m cable. Insole calibration was performed once for each subject as per manufacturers' guidelines. This calibration involved subjects initially walking > 20 steps to allow the insole adjust to conditions in the shoe. The insole was then loaded with total body weight for 1 second by lifting the left foot off the ground, simulating the magnitude and speed of stance phase loading during gait (Figure 2). The same insole was used for each individual for each of his or her walking trials. Because of natural step-to-step variability [18], data from several footfalls was gathered to obtain a representative profile of the subject's foot. Plantar pressure data was collected over 10 metres at a frequency of 50 Hz. Subjects were asked to walk at their normal speed, looking straight ahead. Standardised instructions were given to each subject by the same investigator. Prior to testing, subjects were allowed practice to become comfortable with the procedure. Post-taping, subjects walked around for 2–3 minutes to adapt to the tape. A rest interval between walking trials was offered to all subjects to minimise possible fatigue. Because velocity has been shown to affect plantar pressure values [19], the time taken to complete the walks was also recorded.

### Instrumentation: Motion analysis

Kinematic data was acquired using a CODA mpx64 (Charnwood Dynamics Ltd., Leicestershire, UK) motion analysis system. This system uses a laboratory-based coordinate system, and calculates joint angles based on skin marker positions without the need to define a 'zero' starting position for the rearfoot. The markers were applied by one investigator in line with both manufacturer guidelines and previous research [20]. Markers were positioned on the lateral aspect of the knee joint line in the median frontal plane, the anterior aspect of the lateral malleolus, the posterior inferior lateral aspect of the heel, and the lateral aspect of the fifth metatarsal head. The markers were fixed to the skin with double-sided adhesive tape. The order of testing required removal and immediate replacement of some of these markers when LD tape was being removed prior to analysis of the 'untaped' condition, however the same investigator did this over a very short time period. During testing subjects walked barefoot across a 10-metre walkway at a comfortable 'normal' walking speed. Subjects were instructed to look at a distant mark to prevent them from looking down at the floor. The subject performed 4 gait cycles with the tape and 4 cycles without the tape, since previous research has recommended the use of at least 3 gait cycles to aid reliability [21]. 3D motion data was collected at 200 Hz for 4 seconds while the subject was performing the walks, similar to previous research [20]. Blinding of the data collector regarding subject condition during the testing procedure was not possible.

### Data Analysis

Plantar pressure data from the entire stance phase (heel-strike to toe-off) was collected and analysed using Tekscan software. To avoid any acceleration and deceleration associated with the beginning and end of walks, the middle 3 stance phases of each 10 metre walk were analysed. The foot was divided into a grid with 6 distinct areas to display changes in plantar pressure distribution. The same grid was used for taped and untaped data of each subject, but to accommodate different sized feet, different grids had to be developed for each subject. Insole sensor cells occasionally developed "shorts" where they appeared to be loaded when they are not, and these were edited prior to analysis as per manufacturers' guidelines. Tekscan software calculated the average peak plantar pressure of the middle 3 stance phases in each of the 6 areas. Peak pressure was defined as the highest value recorded by a cluster of 4 cells over the entire stance phase [22,23]. For kinematic data, the stance phase of gait had to be identified in the absence of a force plate to demarcate stance and swing phases. Therefore heel strike was identified using the lowest vertical component of the heel marker and verified with the stick figure diagram [20]. Kinematic data was calculated and analysed by CODA software, before being extracted and entered into Microsoft Excel and averaged for all subjects. The kinematic data analysed included the following parameters at the subtalar joint during the stance phase of gait;

• minimum displacement value, which indicated peak pronation.

• maximum displacement value, which indicated peak supination.

• total displacement which represented total subtalar joint ROM.

• mean displacement value, which indicated mean joint position during stance.

These kinematic values are as defined by the manufacturers and other researchers [20].

### Statistical Analysis

Statistical analysis was undertaken using SPSS 13.0 for Microsoft Windows (Chicago, IL). Data distribution was determined visually using histograms and using the Kolmogornov-Smirnov statistical test. Kinematic data, with the exception of minimum (pronation) values was normally distributed. Plantar pressure data, along with the pronation values from motion analysis, were non-normally distributed. Paired t-tests were carried out on normally distributed data to test for statistically significant differences between taped and untaped conditions. Wilcoxon-Signed Rank tests were carried out on non-normally distributed data to test for significant differences between taped and untaped conditions. The level of significance was set at p < 0.05. The standard error of measurement (SEM) was calculated in line with previous research [24].

## Results

### Demographic Data

20 subjects (6 M, 14 F) met the inclusion criteria. Their mean (+/- SD) age was 22.1 (+/- 5) years.

### Plantar pressure data

**Figure 3:**
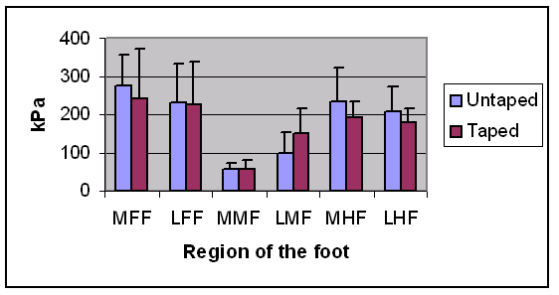
Peak plantar pressure values for taped and untaped conditions for each region of the foot; medial forefoot (MFF), lateral forefoot (LFF), medial midfoot (MMF), lateral midfoot (LMF), medial hindfoot (MHF) and lateral hindfoot (LHF). These differences were statistically significant for the lateral midfoot (p = 0.000), the medial forefoot (p = 0.014), and the medial (p = 0.000) and lateral (p = 0.007) hindfoot.

LD taping resulted in statistically significant increases in peak plantar pressure in the lateral midfoot (p = 0.000), along with significant decreases in pressure in the medial forefoot (p = 0.014), and the medial (p = 0.000) and lateral hindfoot (p = 0.007) (Table 2). No significant changes occurred in the medial midfoot (p = 0.794) or lateral forefoot (p = 0.654) (Figure 3). The actual differences in peak plantar pressure values between taped and untaped conditions for all 6 areas of the foot are also detailed for each subject (see additional file 1).

**Table 2:** Mean (+/- SD) values for peak plantar pressure in taped and untaped conditions for each region of the foot; medial forefoot (MFF), lateral forefoot (LFF), medial midfoot (MMF), lateral midfoot (LMF), medial hindfoot (MHF) and lateral hindfoot (LHF).

	**Untaped**	**Taped**
**MFF**	276.85 (+/- 79.66)	241.5 (+/- 131.44)*
**LFF**	230.65 (+/- 105.43)	227.75 (+/- 108.90)
**MMF**	57.2 (+/- 15.83)	58.7 (+/- 23.17)
**LMF**	99.4 (+/- 52.79)	149.35 (+/- 65.79)*
**MHF**	234.85 (+/- 88.20)	192.05 (+/- 43.05)*
**LHF**	208 (+/- 63.73)	180.2 (+/- 35.49)*

*indicates the difference was statistically significant (p < 0.05)

### Kinematic data

**Figure 4:**
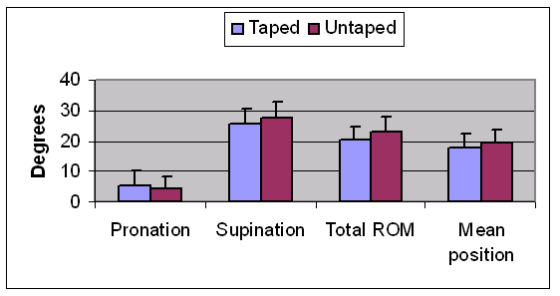
Kinematic values for pronation, supination, total range of motion (ROM) and mean joint position for taped and untaped conditions. These differences were statistically significant for pronation (p = 0.006), supination (p = 0.025) and total ROM (p = 0.000).

The means and standard deviations for pronation, supination, total ROM and joint position under both taped and untaped conditions are displayed in table 3 and figure 4. There was a statistically significant reduction in both pronation (p = 0.006) and supination (p = 0.025) when LD taping was applied. As a result, there was also a significant reduction in total ROM after the application of LD tape (p = 0.000). However the mean rearfoot position was not significantly different between the test conditions (p = 0.188). The actual differences in kinematic values between taped and untaped conditions are also detailed for each subject (see additional file 2).

**Table 3:** Mean (+/- SD) values for pronation, supination, total range of motion (ROM) and mean joint position for taped and untaped conditions.

	**Taped**	**Untaped**
**Pronation #**	5.54 (+/- 4.27)	4.15 (+/- 3.76)*
**Supination**	25.69(+/- 4.06)	27.56 (+/- 4.30)*
**Total ROM**	20.15(+/- 3.64)	23.41 (+/- 3.92)*
**Mean Position**	18.05(+/- 3.50)	19.16 (+/- 3.48)

*indicates the difference was statistically significant (p < 0.05). # lower values for pronation represent increased pronation, and not reduced pronation.

### Data reliability

We did not perform a test-retest reliability study, which significantly limits interpretation of the reliability of the data. Instead, we used the actual study data to calculate values for the SEM, to give an approximate representation of the reliability of the data. Data for plantar pressure could not be used to generate a value for SEM. Kinematic data from each of the four trials was however analysed to obtain values for the SEM of the CODA system (see additional file 3).

## Discussion

The findings of this study suggest that LD taping results in reduced rearfoot motion, and changes in plantar pressure patterns, in a small sample of healthy subjects. In agreement with previous trials, LD taping resulted in an immediate short-term reduction in pronation [3,6,8-10,12]. This is the first trial that has shown this reduction in pronation to be present when measured by both plantar pressure and 3D motion analysis. Interestingly rearfoot motion in general, rather than simply pronation, appears to have been reduced with LD taping. This has not been reported previously, but is consistent with similar research demonstrating that addition of foot orthotics resulted in an overall reduction in rearfoot motion, rather than simply reduced pronation [11]. This suggests that it may be inappropriate to refer to LD taping as 'anti-pronation' taping, as its effects are not solely on pronation ROM. In a wider context, this is important because the technique is used by up to 73% of physiotherapists [25], and it is commonly described as an 'anti-pronation' taping technique, with less consideration of it's effect on supination ROM [26].

### Plantar pressure

Results of the current study indicate that taping caused a significant increase in peak plantar pressure in the lateral midfoot, no change in the medial midfoot or lateral forefoot, and significant decreases in the hindfoot (medial and lateral) and the medial forefoot. Since peak plantar pressures are located more medially in excessively pronated feet [27], these results imply that there may be a trend towards reduced pronation in the midfoot and forefoot, but not in the hindfoot. The results are broadly in accordance with the results of previous similar studies [3,12]. Russo and Chipchase [3] found very similar results in the midfoot and forefoot, however they reported contradictory findings in the hindfoot, where peak pressure was increased after taping. Lange et al. [12] also agreed with the results of the current study, showing a significant increase in lateral midfoot pressure and a reduction in hindfoot pressure after LD taping. Vincenzino et al. [5] also demonstrated a significant reduction in hindfoot contact as well as a non-significant increase in lateral midfoot contact after 'augmented' LD taping, similar to the current study. They examined plantar contact area however, rather than plantar peak pressure. The slight inconsistencies between trials may be explained by differences in the pressure-sensor system used, as well as variations in the exact type of LD taping applied. Despite this, the changes observed in the current study are broadly consistent with those described in the literature.

### Kinematics

Maximum pronation was found to decrease significantly (p < 0.05) as a result of LD taping. This finding is in agreement with results found in other studies [5,7-10,26]. The populations studied in these other trials, and the taping techniques used, were similar to those of the current study. Different outcome measures were however used in previous trials, with the majority being related to measures such as ND and VNH [9,10]. This is the first study examining the effect of LD taping on rearfoot motion using more complex 3D analysis, however the findings regarding reduced pronation are in line with previous studies. The findings of a reduction in supination are interesting in that they appear to indicate that LD taping results in a general decrease in mobility of the rearfoot, rather than having a purely 'anti-pronation' effect, as has typically been described in the literature [7,26]. This is further highlighted by the fact that the mean position of the rearfoot during stance did not change significantly between conditions. The observed reduction in overall rearfoot motion has also been described with the use of foot orthotics, albeit using different methods of motion analysis [11,28]. The effects of LD taping and foot orthotics may be similar, however this has not been proven and further research is needed to clarify if the effects seen here with LD taping also occur with foot orthotics. In addition, previous research [29] indicates that ankle taping reduces ankle joint motion in normal subjects. Although the taping technique and joint motion measured differs, their findings are in line with the current study.

### Mechanism of action

The main proposed mechanism behind the clinical effectiveness of LD taping has been that it restricts rearfoot pronation [10], thereby reducing medial loading and increasing lateral loading through the foot [7,26]. The findings of this study agree only in part with this proposal. While pronation was reduced, LD taping did not result in increased supination, but rather reduced supination. The motion of the rearfoot as a whole was reduced, and the mean position through stance did not alter, with LD taping. The changes in plantar pressure imply a reduction in pronation, particularly during loading of the midfoot and forefoot. The plantar pressure data does not inform us sufficiently about supination range however. It may be that LD taping acts as a controller of general foot hypermobility rather than having a specific 'anti-pronation' effect. These hypotheses require further research before being proven however.

### Future research

The evidence suggests that the effects of LD taping are short lived, although the exact length of time it may be effective for is still unclear [6-9]. This study was limited to the short-term effects of LD taping on non-injured subjects. Obviously further research is required to evaluate if these findings are replicated in a painful population, and how long these effects are maintained. Furthermore, research using foot orthotics suggests that when rearfoot motion is reduced significantly, significant changes may also occur more proximally at the knee joint [11]. Further research into the effects of LD taping on motion in other lower limb joints is warranted. Future use of both kinematic and plantar pressure data in studies examining the effects of LD taping may be warranted as the current study results imply that they inform us of related, but different, aspects of the technique.

### Limitations

The main limitation relates to the fact that both 3D motion analysis and plantar pressure systems are known to be linked to variable data output [21,30]. The current study took steps to minimise this variation however, and the degree of variation is similar for both taped and untaped conditions. 3D motion analysis is a relatively new method of analysing the effect of LD taping on rearfoot motion. All surface marking systems carry a certain degree of error when estimating the motion of joints, however the CODA motion analysis system is sufficiently reliable if a number of gait cycles are used, similar to this study [20,21]. It is difficult to compare absolute values of plantar pressure systems across studies, and it is more appropriate to compare plantar pressure distributions under constant conditions, as in this study [31]. Secondly, a strict protocol was followed when using the F-Scan in order to make the procedure reliable. The F-scan system is highly correlated with force platform measures [16] and is sufficiently reliable [15], particularly when a mean of 3 steps is taken as the representative value [16], similar to recommendations for other pressure measurement systems [32]. Thirdly, other factors which could affect validity e.g. walking speed and surface contact [33], were consistent between taped and untaped conditions. The use of footwear was different for each measurement type, but once again this was consistent between taping conditions. Ideally, the measurement of 3D motion and plantar pressure would occur simultaneously to ensure the gait cycle analysed was identical, and the effect of taping could not have changed. The desire to examine in-shoe plantar pressures obviously would not allow visualisation of the skin markers. Therefore, simultaneous data collection was not possible and correlations between changes in kinematics and plantar pressure distribution were neither possible nor appropriate. This potential bias was minimised by gathering multiple cycles for each measurement system, in line with recommendations regarding a suitable number for adequate reliability for each system [16,21]. This resulted in a different number of gait cycles being performed for plantar pressure and motion analysis, however the number of gait cycles did not vary between the taped and untaped conditions. The absence of a force plate also meant the authors had to visually gauge where heel-strike and toe-off occur. This method has, however, been recommended by the manufacturers and been described in previous research [20]. The need to reposition motion analysis skin markers after the removal of LD tape requires that the kinematic results be interpreted with some caution, as there is a small risk that this could have resulted in slight changes in kinematic angles. Similar to some previous LD taping trials [5,7], the reliability of the investigators was not established in the current study, however this is a potential source of error. This is particularly important given the small magnitude of change between conditions and the high variability of the data. The SEM values for kinematic data exceeded the statistically significant difference observed between taped and untaped conditions. Therefore the data should be interpreted with caution, as some of the difference observed between groups could be due to simply measurement error. A clearer indication of the reliability of the study protocol would require a test-retest reliability study to be performed in advance. The sample size is however in line with previous LD taping trials [5,7,9,10,26]. This high level of data variability is commonly noted in studies of plantar pressure, LD taping and lower limb kinematics [5,15,16]. The effect of taping was examined only during the stance phase, due to the fact that maximum pronation has been found to occur during the middle-to-late stance phase of the gait cycle [1], and symptoms are usually related to weight bearing. The size of the change with LD taping was statistically significant, but we cannot say whether this would be clinically significant. We did not examine whether the taping was performed identically for each subject, however one person performed all taping to minimise error and the tape applied did not change between the two measurement techniques. The sample size was small, and a suitable power calculation was not performed due to the exploratory nature of the study, and this limits external validity. Subjects were not randomly selected, but were a sample of convenience. The amount of time subjects were given to become accustomed to the tape varied somewhat between 2 and 3 minutes, which is a potential source of error. Also, there is a very slight risk of a residual effect of taping even after its removal, which could potentially have affected the baseline 'untaped' kinematic data. Finally, neither subjects nor investigators were blinded to taping condition, as this was not feasible.

## Conclusion

While this relatively small study does have some limitations, we believe, as it is the first study to combine 3D kinematic and plantar pressure measurement of the effects of LD taping, that its results are noteworthy. The study demonstrated that LD taping reduced both pronation and supination in the rearfoot during the stance phase of gait in healthy subjects with a ND exceeding 10 mm. LD taping also significantly altered the plantar pressure pattern of the foot. Clinically, this may support the use of LD taping in the treatment of symptoms related to increased foot mobility. Despite the description of LD taping as an 'anti-pronation' taping technique, it may work by limiting overall motion at the rearfoot. Further research is needed, particularly in clinical populations and examining the effects of foot orthotics. Further research is also required to establish the effect of reduced rearfoot ROM on other joints of the lower limb and its implications for injured subjects. It is important that future similar studies clarify whether the changes observed are greater than measurement error, which the current study was unable to do. Studies will also need to be conducted to establish the length of time that this effect of LD taping on the rearfoot lasts in a clinical population.

## Competing interests

The authors declare that they have no competing interests.

## Authors' contributions

KOS was involved in conception and design of the study, data analysis and interpretation, as well as drafting and editing the final document for publication. NK was involved in conception and design of the study, data analysis and interpretation, as well as drafting and editing the final document for publication. EON was involved in conception and design of the study, data collection, data analysis, as well as drafting and editing the final document for publication. UNM was involved in conception and design of the study, data collection, data analysis, as well as drafting and editing the final document for publication.

## Funding

None

## Pre-publication history

The pre-publication history for this paper can be accessed here:



## Supplementary Material

Additional file 1 Peak plantar pressure data for all 20 subjects (averaged) in taped and untaped conditions, for each region of the foot; medial forefoot (MFF), lateral forefoot (LFF), medial midfoot (MMF), lateral midfoot (LMF), medial hindfoot (MHF) and lateral hindfoot (LHF). Click here for file

Additional file 2 Kinematic data for all 20 subjects (averaged) in taped and untaped conditions. Click here for file

Additional file 3 Estimated standard error of measurement (SEM) values (in degrees) for kinematic data, for both taped and untaped conditions. Click here for file
