# Supplementary material for: The effect of low-dye taping on rearfoot motion and plantar pressure during the stance phase of gait
Source: BMC Musculoskelet Disord. 2008 Aug 18;9:111. doi: 10.1186/1471-2474-9-111 (PMC2529302; doi:10.1186/1471-2474-9-111)
Supplement: Additional file 2 — Kinematic data for all 20 subjects (averaged) in taped and untaped conditions. [file 1471-2474-9-111-S2.doc]

| Subject | Mean position | | Supination | | Pronation | | Total ROM | |
| --- | --- | --- | --- | --- | --- | --- | --- | --- |
|  | Taped | Untaped | Taped | Untaped | Taped | Untaped | Taped | Untaped |
| 1 | 14.135 | 18.0575 | 20.2175 | 25.6725 | 5.335 | 4.0875 | 14.8825 | 21.585 |
| 2 | 18.85 | 23.52 | 24.85 | 30.255 | 6.16 | 4.05 | 18.69 | 26.205 |
| 3 | 14.12 | 16.1125 | 21.875 | 24.4725 | 2.2975 | 3.1425 | 19.5775 | 21.33 |
| 4 | 23.185 | 25.49 | 30.1675 | 35.765 | 10.8325 | 11.7575 | 19.335 | 24.0075 |
| 5 | 15.0225 | 15.7375 | 22.62 | 20.2675 | 2.38 | -1.8725 | 20.24 | 22.14 |
| 6 | 17.245 | 18.4775 | 23.76 | 23.265 | 5.5425 | 3.6475 | 18.2175 | 19.6175 |
| 7 | 8.39 | 16.755 | 14.6 | 23.105 | -2.24 | 4.355 | 16.84 | 18.75 |
| 8 | 19.5825 | 18.7175 | 27.3525 | 26.1075 | 10.4275 | 4.9825 | 16.925 | 21.125 |
| 9 | 16.155 | 17.1225 | 24.6125 | 26.24 | 2.2725 | 1.115 | 22.34 | 25.125 |
| 10 | 18.2775 | 23.635 | 25.92 | 30.515 | 4.23 | 2.7975 | 21.69 | 27.7175 |
| 11 | 17.465 | 22.6425 | 26.7425 | 29.7975 | 10.0525 | 5.6175 | 16.69 | 24.18 |
| 12 | 21.095 | 21.42 | 30.0775 | 32.785 | 3.0725 | 3.4 | 27.005 | 29.385 |
| 13 | 22.5525 | 16.82 | 32.24 | 27.1675 | 9.525 | 9.5075 | 22.715 | 17.66 |
| 14 | 20.7225 | 23.815 | 30.065 | 32.4275 | 8.62 | 6.5675 | 21.445 | 25.86 |
| 15 | 15.0425 | 12.8575 | 22.7775 | 21.465 | -3.01 | -5.165 | 25.7875 | 26.63 |
| 16 | 19.055 | 19.02 | 26.5975 | 30.6825 | 7.8 | 5.995 | 18.7975 | 24.6875 |
| 17 | 18.88 | 17.605 | 26.715 | 25.865 | 2.8025 | 1.415 | 23.9125 | 24.45 |
| 18 | 20.7875 | 14.0475 | 25.7525 | 22.6125 | 12.7425 | 8.8725 | 13.01 | 13.74 |
| 19 | 20.1125 | 19.8325 | 28.4725 | 31.055 | 3.6125 | 2.81 | 24.86 | 28.245 |
| 20 | 20.4325 | 21.4325 | 28.4375 | 31.7125 | 8.3825 | 5.9 | 20.055 | 25.8125 |
| AVG | 18.05538 | 19.15588 | 25.69263 | 27.56175 | 5.541875 | 4.149125 | 20.15075 | 23.41263 |
