# Supplementary material for: The effect of low-dye taping on rearfoot motion and plantar pressure during the stance phase of gait
Source: BMC Musculoskelet Disord. 2008 Aug 18;9:111. doi: 10.1186/1471-2474-9-111 (PMC2529302; doi:10.1186/1471-2474-9-111)
Supplement: Additional file 3 — Estimated standard error of measurement (SEM) values (in degrees) for kinematic data, for both taped and untaped conditions. [file 1471-2474-9-111-S3.doc]

|  | Pronation | Supination | Mean Position |
| --- | --- | --- | --- |
| Taped | 1.948 | 2.555 | 2.442 |
| Untaped | 1.563 | 3.223 | 3.327 |
