# Supplementary material for: The effect of low-dye taping on rearfoot motion and plantar pressure during the stance phase of gait
Source: BMC Musculoskelet Disord. 2008 Aug 18;9:111. doi: 10.1186/1471-2474-9-111 (PMC2529302; doi:10.1186/1471-2474-9-111)
Supplement: Supplementary file 3 [file 1471-2474-9-111-S1.doc]

| Subject | MFF | | LFF | | MMF | | LMF | | MHF | | LHF | |
|---|---|---|---|---|---|---|---|---|---|---|---|---|
| | *Untaped* | *Taped* | *Untaped* | *Taped* | *Untaped* | *Taped* | *Untaped* | *Taped* | *Untaped* | *Taped* | *Untaped* | *Taped* |
| 1 | 239 | 145 | 293 | 339 | 56 | 27 | 77 | 191 | 245 | 223 | 231 | 204 |
| 2 | 261 | 251 | 168 | 167 | 54 | 55 | 76 | 87 | 210 | 200 | 208 | 204 |
| 3 | 290 | 158 | 102 | 93 | 58 | 54 | 43 | 76 | 197 | 207 | 147 | 161 |
| 4 | 197 | 255 | 119 | 120 | 68 | 51 | 63 | 79 | 227 | 221 | 178 | 178 |
| 5 | 319 | 670 | 284 | 289 | 55 | 40 | 64 | 211 | 546 | 250 | 339 | 238 |
| 6 | 281 | 294 | 218 | 352 | 40 | 43 | 70 | 170 | 255 | 228 | 257 | 208 |
| 7 | 382 | 388 | 281 | 361 | 61 | 52 | 169 | 228 | 233 | 205 | 195 | 197 |
| 8 | 422 | 328 | 337 | 324 | 68 | 126 | 79 | 163 | 262 | 256 | 188 | 194 |
| 9 | 178 | 101 | 133 | 168 | 48 | 51 | 61 | 127 | 239 | 142 | 138 | 146 |
| 10 | 283 | 299 | 281 | 255 | 50 | 57 | 246 | 248 | 233 | 236 | 250 | 215 |
| 11 | 388 | 343 | 519 | 486 | 46 | 89 | 102 | 157 | 375 | 219 | 383 | 227 |
| 12 | 208 | 186 | 184 | 176 | 48 | 60 | 69 | 81 | 227 | 204 | 225 | 174 |
| 13 | 260 | 173 | 178 | 209 | 60 | 34 | 59 | 127 | 204 | 172 | 174 | 144 |
| 14 | 222 | 189 | 154 | 168 | 102 | 96 | 103 | 106 | 145 | 127 | 136 | 119 |
| 15 | 106 | 83 | 217 | 196 | 38 | 52 | 207 | 271 | 221 | 210 | 232 | 216 |
| 16 | 250 | 213 | 159 | 163 | 29 | 33 | 120 | 128 | 152 | 148 | 155 | 149 |
| 17 | 313 | 225 | 327 | 338 | 72 | 63 | 133 | 263 | 186 | 103 | 214 | 118 |
| 18 | 229 | 213 | 118 | 131 | 69 | 71 | 98 | 96 | 163 | 131 | 158 | 144 |
| 19 | 391 | 234 | 168 | 135 | 73 | 66 | 82 | 94 | 189 | 185 | 179 | 203 |
| 20 | 318 | 82 | 373 | 85 | 49 | 54 | 67 | 84 | 188 | 174 | 173 | 165 |
| **Avg** | 276.85 | 241.5 | 230.65 | 227.75 | 57.2 | 58.7 | 99.4 | 149.4 | 234.85 | 192.1 | 208 | 180.2 |
